# The Role of the Microbiome in Cancer Initiation and Progression: How Microbes and Cancer Cells Utilize Excess Energy and Promote One Another’s Growth

**DOI:** 10.1007/s13668-019-0257-2

**Published:** 2019-02-13

**Authors:** Corrie M. Whisner, C. Athena Aktipis

**Affiliations:** 1 College of Health Solutions, Arizona State University, Phoenix, AZ USA; 2 Department of Psychology, Center for Social Dynamics and Complexity, Center for Evolution and Medicine, Biodesign Institute, Arizona State University, PO Box 871104, Tempe, AZ 85287-1104 USA

**Keywords:** Cancer, Neoplasms, Neoplastic processes, Metastasis, Cell proliferation, Microbiome, Microbiota, Microbe, Diet, Nutrition, Western diet, Caloric restriction, Immune system, Inflammation, Ecology

## Abstract

**Purpose of Review:**

We use an ecological lens to understand how microbes and cancer cells coevolve inside the ecosystems of our bodies. We describe how microbe-cancer cell interactions contribute to cancer progression, including cooperation between microbes and cancer cells. We discuss the role of the immune system in preventing this apparent ‘collusion’ and describe how microbe-cancer cell interactions lead to opportunities and challenges in treating cancer.

**Recent Findings:**

Microbiota influence many aspects of our health including our cancer risk. Since both microbes and cancer cells rely on incoming resources for their survival and replication, excess energy and nutrient input from the host can play a role in cancer initiation and progression.

**Summary:**

Certain microbes enhance cancer cell fitness by promoting proliferation and protecting cancer cells from the immune system. How diet influences these interactions remains largely unknown but recent evidence suggests a role for nutrients across the cancer continuum.

## Introduction

Microbes play an important role in human health and disease, including influencing our cancer risk. Microbes and cancer cells coevolve inside the ecosystems of our bodies, and both rely on incoming resources for their survival and replication. This means that what we eat—in particular, whether we have excess energy and nutrients—can affect the growth of both cancer cells and microbial cells. In addition, cancer cells and microbes can influence each other’s replication and survival through the production of factors. Taken together, these facts suggest that the interactions between cancer cells and microbial cells may be very important in cancer initiation and progression.

The burden of cancer is undeniably high—1,735,350 newly diagnosed cases and 609,640 deaths were estimated for 2018 in the USA alone, and more than 38% of people develop cancer during their lifetime [1]. With the increasing prevalence of cancer comes an increased economic burden with direct medical costs reaching $80.2 billion in 2015 [2]. These costs will continue to rise as 23.6 million new cases of cancer are expected by 2030 [2].

Recently, the gut microbiome has emerged as an important mediating factor of health and disease [3]. We have approximately as many microbes in and on us as we have human cells (3.8 × 10^13^ microbial cells relative to 3.0 × 10^13^ human cells [4]). The human gut houses the most diverse and metabolically varied proportion of these microbes when compared to any other body surface, serving as home to more than 1000 unique species. These species express 3.3 million genes—orders of magnitude more than the 23,000 expressed human genes [5]. Interactions between microbes and human cells play important roles in human metabolism including digestion of complex carbohydrates, production of essential amino acids, creation of beneficial fatty acids and vitamin compounds, and degradation of xenobiotics including environmental toxins and medications [6]. During cancer progression, these metabolic interactions between microbes and human cells may shift from ones that support health to ones that threaten it, as microbes begin interacting with cancer cells rather than healthy human cells. Indeed, microbial dysbiosis has been found to contribute to gastrointestinal cancer development [7].

It is clear that microbes play important roles in obesity, gastrointestinal, and cardiometabolic disease prevention and treatment [8], suggesting that microbes alter human metabolism. However, there are still many open questions about how microbes and cancer cells interact metabolically and how these processes contribute to cancer. In this review, we evaluate the current literature on microbiota in cancer risk, discuss how gene-environment interactions contribute to microbial mechanisms of cancer, explore how diet influences cancer risk via the microbiome, and describe how cooperative interactions between microbes and cancer cells may influence cancer.

## Microbes Contribute to Cancer Risk

The human gut is a diverse ecosystem including fungi, bacteria, viruses, and archaea, of which bacteria from the phyla Firmicutes, Bacteroidetes, Proteobacteria, and Actinobacteria are most prevalent [9]. Microbes can help maintain the intestinal barrier which keeps potentially harmful organisms from reaching the epithelium where they can cause illness and injury [10]. Currently, microbes are believed to contribute to cancer risk by modifying DNA in human somatic cells thereby altering cell cycle controls, accelerating cell proliferation, and disrupting normal programs for controlled cell death that protect the body from aberrant cells.

Microbes have been linked to approximately 10–20% of human cancers [11]. To date, ten microorganisms have been designated as carcinogens by the International Agency for Cancer Research, one of which is *Helicobacter pylori* for its association with stomach cancer [11]. Despite observed links to cancer, these microbes reside in a large proportion of the human population, many of whom never develop cancers associated with these otherwise commensal microorganisms.

## Microbes and Cancer Cells Evolve in the Ecology of the Body and in the Tumor Microenvironment

Our bodies are essentially ecosystems in which cells can evolve. As in any ecosystem, the players that best survive and replicate end up making up a larger proportion of the next generation in the population—this is the process of evolution via natural selection. Cancer is fundamentally a problem of cells evolving in the body to proliferate quickly, monopolize resources and evade cellular controls that otherwise make the body function normally [12]. Similarly, diseases caused by harmful microbes are the result of microbes overproliferating, monopolizing metabolic resources, and producing virulence factors that interfere with normal organismal functioning [13], thereby allowing further microbial imbalances (dysbiosis) to occur.

Cancer cells not only evolve inside the ecosystem of the body, they also can create a microenvironment around the tumor that facilitates their growth [14, 15]. This tumor-promoting microenvironment has growth factors, angiogenic signals (signal growth of blood vessels that feed tumors) and fibroblast ‘support’ cells [14]. The microenvironment can promote the tumor, but earlier in progression, it can also be an important part of limiting the tumor. If tissue homeostasis is functioning properly and the immune system has not yet become dysregulated, then the microenvironment may help to suppress cancer [16–18].

More generally, the immune system is an important aspect of the ecology around tumors. Normally, the immune system monitors the tissues of the body for pathogens and cancer cells, targeting harmful cells for destruction. This process of immune predation helps keep potentially harmful cells from damaging healthy human tissues. However, cancer cells and pathogens can also evolve to evade the immune system just like prey evolves to evade predators [19]. Additionally, other aspects of the immune response (e.g., inflammation accompanying wound healing) can be co-opted by cancer cells and pathogens to generate a proinflammatory environment in which both cancer and pathogen cells can thrive [14, 17].

Tumor microenvironments can include microbes that reside in or near the tumor. Microbes can alter the microenvironment by producing factors that influence cancer cells. For example, certain strains of *E. coli* produce colibactin toxin that is more commonly found in the mucosa of individuals with colorectal cancer than healthy controls [20]. Colibactin induces cells in the microenvironment to produce growth factors which may promote tumor growth [21]. Another way that microbes can influence the microenvironment is through producing bacterial biofilms which have been associated with higher cell proliferation rates and increased risk of colorectal cancer [22]. The tumor microenvironment in gastrointestinal tissue can be influenced by all of the microbes and nutrients that are present. Therefore, what and how much we eat can have downstream impacts on the ecology of gastrointestinal tissues in ways that promote or limit cancer.

## Excess Energy Can Feed Both Cancer Cells and Harmful Microbes

Microbial community structure (diversity and abundance of specific taxa) and function is rapidly influenced by acute [23] and long-term dietary changes [24–26], thereby highlighting the importance of dietary inputs for gut microbial community growth and maintenance. Consumption of high-fat or Western style diets has been associated with cancer [27, 28]. Further, obesity as a result of excess caloric consumption has been linked with increased cancer risk [29]. This link between excess energy consumption and cancer may be mediated by gut microbial metabolism. Microbiome transfers from obese murine donors to germ-free counterparts has been shown to lead to weight gain and fat deposition in recipient mice [30, 31]—even when mice have the same dietary inputs [30, 32].

The links between cancer and obesity are clear [33], and some work suggests that microbes may play an important role in this link. Caloric restriction studies suggest that cancer outcomes are improved via increased gut microbial diversity and subsequent reductions in inflammation [34•, 35•, 36, 37]. This may be mediated by improved gut barrier integrity which minimizes translocation of microbial-derived inflammatory markers including lipopolysaccharide and also beneficial shifts in microbial abundance (e.g., increased abundance of *Lachnospiraceae*), as observed in obese women on very low calorie diets (~ 800 kcal/day) [34^•^].

As with any ecological system, the introduction of excess resources can disrupt the normal interactions among the constituents in that ecosystem. In the case of the human body, introducing excess energy can disrupt the interaction between the microbes and human somatic cells. Under ideal conditions, growth and proliferation of microbes and our somatic cells is limited by access to resources like carbohydrates, protein and fat, as well as through somatic cell cycle controls. Glucose is a preferred fuel source for most human cells, from which cellular energy in the form of ATP is used to fuel cellular processes. In the case of cancer, this otherwise oxygen-requiring pathway switches to less efficient processes such as fermentation. This metabolic disruption, referred to as Warburg metabolism [38], results in the production of lactate which destroys the extracellular environment and facilitates invasion into new tissues and metastasis. Future work should investigate whether these metabolic shifts also encourage translocation and growth of lactate-utilizing microbes from the intestinal tract [39] to tumor regions. Interestingly, there is some evidence that microbes that thrive in anaerobic conditions may actually suppress replication of cancer cells, though the mechanisms of action remain unknown [40].

**Fig. 1:**
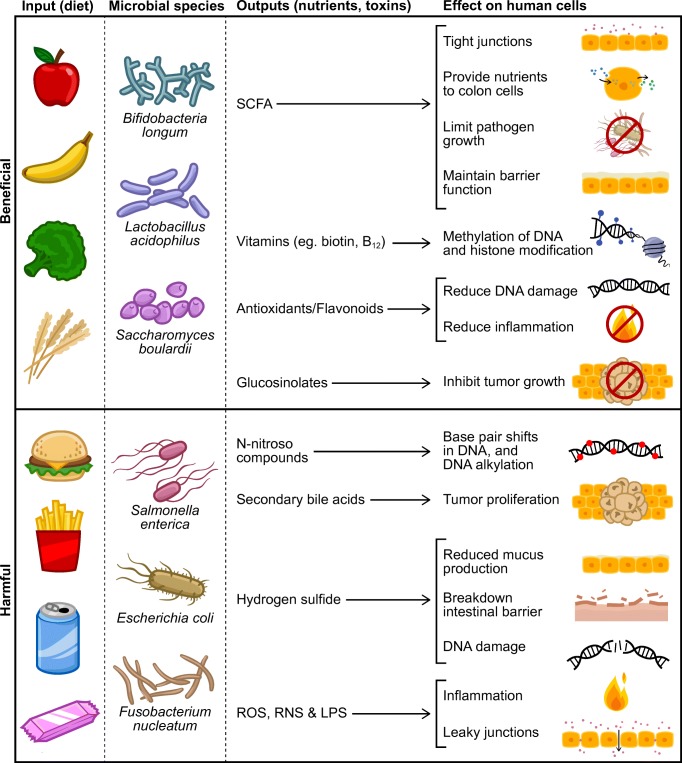
Important metabolites and associated mechanisms that promote and inhibit the collusion of gut microbes and cancer cells. LPS, lipopolysaccharide; ROS, reactive oxygen species; RNS, reactive nitrogen species; SCFA, short-chain fatty acids

## Dietary Modification Can Influence Microbial and Cancer Cell Growth

The introduction of agrarian (farmers and pastorals) lifestyles 10,000 years ago followed by the industrial revolution led to the emergence of a genotype more adept at processing complex carbohydrates from plant-based foods, rather than the high-protein diets of hunter-gatherers [41]. Our ancestors relied on their gut microbes to break down plant fiber so that their bodies could obtain adequate amounts of energy and nutrients. Gut microbiota express enzymes that carry out diverse reactions, including fermentation, hydrolysis, denitrification, sulfate reduction, and aromatic fission, to process compounds that persist in the gastrointestinal tract and are not metabolized by human enzymes. Today, we have access to plentiful simple and complex carbohydrates, in addition to various other foods that our bodies process with little assistance from microbes. This evolutionary mismatch—especially with regard to increased sugar consumption—appears to contribute to cancer risk [42].

### Fruits, Grains, and Vegetables

Various sugars, starches, dietary fibers, and polyphenolic compounds are present in fruits, grains, and vegetables. Regular consumption of these plant-based foods has been associated with cancer prevention [43] (Fig. 1). Many of the plant components associated with improved health have been attributed to the gut microbiota and their ability to metabolize otherwise non-digestible plant stuffs into bioactive compounds including short-chain fatty acids and bioactive phytochemicals.

Short-chain fatty acids (acetate, propionate, butyrate) are byproducts of the fermentation and hydrolysis of complex carbohydrates and dietary fiber that reach the colonic microbiota [44]. Short-chain fatty acid (SCFA) effects begin at the intestine level, where butyrate is utilized as a primary fuel source for enterocytes, and extend to systemic influences where butyrate and propionate regulate glucose and lipid metabolism in the liver [45]. Butyrate also induces cell differentiation, apoptosis, and histone hyperacetylation [46, 47]. While these effects are believed to limit cancer initiation and progression, the effects of butyrate seem to depend on host genotype and SCFA concentrations. In a mouse model of colorectal cancer, in which cancer cells preferentially utilized glucose, butyrate was forced to accumulate in the nucleus where it ultimately increased histone acetylation and subsequent apoptosis thereby decreasing cancer cell proliferation [48]. Conversely, in mice with mutations in the Msh2 gene, involved in mismatch repair, microbiota-derived butyrate enhanced tumor cell proliferation [49]. Excess production of acetate in the gut has been linked to altered insulin regulation and obesity [50], and excess energy production via microbial conversion of fiber to SCFA may contribute to obesity and cancer, for which dietary intake is an important mediator [45, 51].

Consumption of plant polyphenols, flavonoids [52, 53] and glucosinolates [54, 55], is associated with reduced cancer risk. Specific microbes can convert glucosinolates from cruciferous vegetables into isothiocyanates which have anticancer properties. These microbes include *Eggerthella spp.*, *Alistipes putredinis*, *Eubacterium hallii*, and *Phascolarctobacterium faecium* [56], of which *Eggerthella* and *Alistipes* also degrade starch and dietary fibers [57]. Fiber-poor diets, which often lack polyphenols, enhance microbial pathogenicity and degrade barrier function via loss of intestinal mucus in animal models [58•], but how this relates to cancer risk remains understudied. A recent meta-analysis suggests that flavonoids, quercetin and apigenin, may reduce the odds of colon cancer [53]. Soy isoflavones have also been implicated in both anticancer and tumor-promoting pathways [59] which may be dependent on which downstream metabolites and nutrients are available to gut microbiota [60].

### Protein- and Fat-Containing Foods

Proteins and amino acids can also be metabolized into phenols, indoles, ammonia, amines, sulfur compounds, and other organic acids [61]. These products are the result of hydrolysis, deamination, decarboxylation, fermentation and elimination reactions. Fatty acids and other lipids can also be metabolized by gut microbes, largely in the conversion of primary to secondary bile acids. These conversions alter both the gut microbiome as well as liver signaling [62], and have also been identified as carcinogenic compounds [63, 64].

High-protein and high-fat diets have been shown to increase the production of potentially carcinogenic branched chain fatty acids, secondary bile acids, and N-nitroso compounds [65, 66] (Fig. 1). Animal product-rich diets that enhance secondary bile acid production promote the presence of bile-tolerant microbes, while decreasing the abundance of plant polysaccharide-metabolizing microbes [23]. The lower the carbohydrate content paired with high-protein diets, the lower the butyrate production and beneficial *Roseburia/Eubacterium rectale* abundance in feces [65]. Reductions in animal product consumption have also been associated with a lower risk of colon cancer than increasing consumption of fiber [67]. Together, these facts suggest that consuming adequate carbohydrates while avoiding excess protein may be a key to reducing cancer risk.

## Microbes Can Promote Cancer Through Various Mechanisms

Ideally, normal human cells and commensal microbes cooperate to keep us healthy: we provide resources for microbes, microbes metabolize nutrients for us; we provide them with an environment to live in, they help protect us from invading pathogens [13]. In cancer, this cooperation between normal cells and beneficial microbes may break down, resulting in dysbiosis. In some cases, the cooperation between normal cells and beneficial microbes may be replaced by cooperation between cancer cells and harmful microbes.

Both microbes and cancer cells evolve in the body’s ecosystem. Thus, microbes and cancer cells that interact with one another in ways that increase their proliferation and enhance their ability to avoid detection by the immune system can potentially gain an evolutionary advantage over those that do not interact in mutually beneficial ways. In other words, selection in the body may sometimes favor microbes and cancer cells that cooperate in order to gain an evolutionary advantage over non-cooperators, with cooperation being stabilized by the evolutionary mechanisms of positive assortment [68] and/or partner choice [69, 70]. In this section, we describe several mechanisms by which microbes and cancer cells can potentially enhance one another’s evolutionary fitness, including altering one another’s rates of proliferation and survival. We also discuss how microbes and cancer cells could come to have high fitness interdependence [71] if they create and thrive within similar microenvironments.

### Microbes Can Damage Cell DNA, Initiating Cancer

Microbes can produce genotoxins which damage DNA. For example, colibactin produced by *E. coli* and Enterobacteriaceae induces double-strand breaks in host cell DNA [72]. Microbes also produce free radicals that damage DNA [73]. *B. fragilis* produces reactive oxygen species that can damage host DNA and contribute to colon cancer [74].

### Microbes Can Increase Cancer Cell Proliferation

*Helicobacter pylori* has been well studied as a bacterial strain with links to cancer. Initially, *H. pylori* was found to increase cell proliferation and induce tumors in Mongolian Gerbils [75]. Twelve human case-control studies further support this microbe-cancer link with data suggesting that *H. pylori* detection was a strong risk factor (odds ratio of 3.0) for gastric adenocarcinoma [76]. Interestingly, *H. pylori* colonizes stomachs of approximately 50% of the world’s population but only a minority of these individuals develop gastric cancer [77]. This may depend on carcinogenic phenotypes of the host which allow *H. pylori* to persist and induce cancer proliferation in the stomach [78••] through virulence factor CagA which stimulates cell proliferation [79].

### Cancer Cells and Microbes Can Provide Growth Factors for Each Other

Senescent cells (cells no longer dividing) can secrete growth factors into the nearby cellular environment to further growth of surrounding epithelial tissues. Bacteria capable of producing the toxin colibactin may mediate this signaling pathway. Specifically, *E. coli* production of colibactin is thought to induce the release of growth factors that promote tumor growth [21, 80].

### Cancer Cells and Microbes May Protect One Another from the Immune System

Commensal microbes in the gut are involved in complex interactions with the human immune system. Chronic inflammation as a result of bacterial infections with *H. pylori*, *Campylobacter jejuni*, and *Chlamydia psittaci* can result in lymphomas that are mediated by overactive adaptive and innate immune signaling [81–83]. Enterotoxigenic *B. fragilis* has been linked to colon cancer in mice via inflammatory pathways that involve Stat3 signaling and increases in IL-17-secreting CD4^+^ T cells [84]. Microbes also interfere with the ability of the immune system to detect them through a variety of different mechanisms, including interfering with natural killer cell activity [85], which could have an impact on the ability of the body to detect and respond to cancer cells.

### Microbes Can Increase Cancer Risk by Altering the Intestinal Barrier and Associated Biofilms

The mucosal lining provides protection to the host from invading pathogens and also provides an environment to beneficial microbes that helps to cultivate them. When the mucosal lining is broken down, as happens with infection by harmful microbes, this can contribute to a pro-cancer environment with greater inflammation [86].

Some bacteria excrete polymers that can form the basis for biofilms. These biofilms create microenvironments for microbes that provide benefits including protection from external threats and access to resources/nutrients [87]. Biofilms are sometimes found in the colon, and they are often associated with colorectal cancer, particularly on the right side of the colon [22], which is the first portion of the colon that early-stage feces travel through. Tissues with biofilms on them have been associated with greater permeability of the intestinal barrier and greater immune activation [22].

### Microbes Can Induce Host Cell Proliferation Which Can Expand Microbes’ Ecological Niche

Many microbes can use host cells to expand their ecological niches through inducing proliferation of the cells upon which they rely. For example, viruses replicate themselves after entering the nucleus, integrating with host cell DNA and inducing cell proliferation. This is the mechanism underlying virally initiated cancers such as HPV [88]. Bacteria can also expand their ecological niches through inducing cell proliferation. *Fusobacteria* does this by entering the cell and inducing proliferation [89], which expands the ecological niche for the microbes [85]. This also has the effect of promoting colorectal cancer. Microbes that bind to epithelial surfaces then increase the proliferation rates of the cells they are attached to like *H. pylori* does to gastric cells [85]. In these situations, harmful microbes and cancer cells may have aligned fitness interests. Such fitness interdependence can arise anytime entities can benefit from one another’s success [71], as is the case when microbes benefit from the expansion of the cancer cell population because this increases the ecological niche for microbes.

### Microbes Can Prompt Cells to Shift to a More Metastatic Phenotype

One of the key steps in the transition from a benign neoplasm to a malignant cancer is the epithelial to mesenchymal transition (EMT), where cells transform from mostly stationary epithelial cells to motile mesenchymal cells. A number of microbes, including *Bacteroides fragilis*, *Fusobacterium nucleatum*, and *Enterococcus faecalis* have been found to produce toxins that contribute to EMT, typically through altering the normal adhesion between cells [90].

### Microbes Produce Quorum Sensing Molecules That Can Contribute to Metastasis

Some microbes, including *Bacillus sp.*, *E. faecium* and *Escherichia coli,* produce peptides that act as quorum sensing molecules (molecules that microbes use to coordinate their gene expression and behavior) that can contribute to metastasis. These quorum sensing molecules appear to alter host epithelial growth factors, which activate intracellular signaling that eventually leads to metastasis [91••].

### Microbes Alter Cancer Cell Epigenetics to Enhance Cancer Cell Proliferation

Microbes are capable of producing metabolites that regulate DNA expression. For example, *Bifidobacterium spp*. produce folate, a major methyl donor in the pathway that leads to S-adenosylmethionine, the metabolite that donates methyls to silence genes. *F. nucleatum*, frequently enriched in patients with colorectal cancer, has been correlated with DNA methylation of genes within the inflamed colonic mucosa which enhance tumorigenesis [92, 93]. Chromatin remodeling via acetylation and deacetylation of histones is also an important aspect of DNA expression regulation. The gut microbiome is thought to play a role in histone acetylation given that butyrate, a metabolite of gut microbes including *Megasphaera*, *Roseburia*, *Faecalibacterium*, *Clostridium*, etc. [94], is a potent inducer of intestinal Treg cell differentiation via histone acetylation [95]. Non-coding RNAs (microRNA, small interfering RNA, and long non-coding RNA) have also been linked to the gut microbiota and host health. Differential expression of long non-coding RNAs has been observed in cells colonized with *E. coli* [96]. Post-transcriptional modifications via altered microRNA expression have been noted after infection with *Salmonella spp.* [97] and *F. nucleatum* [98••], of which *F. nucleatum* is thought to influence colon cancer via autophagy regulation.

## Opportunities and Challenges in Treating Cancer Given Interactions with the Microbiome

### Can We Jump Start the Immune System to Break up Microbe-Cancer Cell Cooperation?

When the immune system is functioning properly, it limits the growth and proliferation of harmful microbes and cancer cells. Microbes and cancer cells may cooperate to create an ecological niche that allows them to proliferate outside of normal immune control. So immune therapies targeted at disrupting microbe-cancer cell interactions may have potential for treatment.

### Can Specific Microbes Be Used to Control Cancer Cell Populations as a Part of Treatment?

Microorganisms, such as *Mycobacterium bovis BCG*, have been used in cancer treatments for more than 100 years, including the successful treatment of bladder cancer [40]. Additionally, many microbial products have been used in cancer treatment, including redox proteins like azurin [40]. The mechanisms of action appear to be diverse, with some activating the immune system, others inducing cell death via apoptosis and others inhibiting the growth of new blood vessels [40], thereby depriving tumors of resources. Future work should investigate the metabolic and ecological interactions between tumor cells and microbes that underlie this effect in order to discover new microbes that can be used in cancer treatments.

### Can Commensal Microbes Enhance the Effectiveness of Therapy?

In mice, having intact commensal microbes leads to greater effectiveness of cancer therapy [99], but other microbes reduce the effectiveness of cancer therapy and increase resistance [98••]. The mechanisms underlying these positive and negative effects on therapy are currently unknown. Future work exploring these interactions could have therapeutic potential.

### Can Foods Containing Prebiotics and Probiotics Help Prevent and Treat Cancer?

While a few cases of adverse events in cancer patients taking probiotics have been published [100], the potential benefits of pre- and pro-biotics observed in pre-clinical models suggest that these bioactive food components may decrease cancer risk by improving intestinal barrier function, immunomodulation, and metabolic and antiproliferative effects [101]. However, prospective longitudinal data are extremely limited in humans. This work is required before making clinical recommendations.

## Conclusion

Human gut microbiota play an important role in enhancing and inhibiting cancer initiation and progression. Dietary intake is an important way of shaping this community but inter-individual variability in microbiome community structure may influence how people respond to different dietary constituents. Not much is known about how dietary inputs affect microbe-cancer cell interactions, but it appears that excess energy inputs may encourage the growth of both cancer cells and pathogenic microbes. While certain microbes can enhance cancer cell fitness by promoting proliferation and protecting cancer cells from the immune system, others may protect against cancer. Nonetheless, there are many open questions with regard to how individual differences in habitual dietary intake, microbial structure and function, and human genetics contribute. Many exciting opportunities for leveraging this new work on microbe-cancer cell interactions to improve prevention and treatment exist. Future work should focus on understanding the complex dynamic interactions between cancer cells and microbes over time both in healthy individuals and cancer patients.
